# B cell directed cytokines

**DOI:** 10.1186/ar3420

**Published:** 2011-09-16

**Authors:** Peter E Lipsky

**Affiliations:** 1 Charlottesville, VA, USA

## 

The prototypic autoimmune disease, systemic lupus erythematosus (SLE), is known to be associated with polyclonal B cell activation [1]. A number of cytokines play essential roles in driving or supporting B cell responses, and are, therefore, candidate targets for controlling the B cell activity in SLE. Among these cytokines are IL-6, IL-21 and BAFF/BLyS. IL-6 is a pleiotropic cytokine with effects on a number of cell types, including B cells, where it plays as essential role in plasma cell differentiation and survival. Blocking IL-6 activity with tocilizumab is approved for treatment of rheumatoid arthritis and preliminary data suggest that it might also be effective in the treatment of SLE [2]. Importantly, treatment of SLE is associated with a decline in anti-DNA antibodies and also a decrease in the frequency of circulating plasma cells, suggesting that at least part of its action relates to an impact on terminal differentiation of B cells into plasma cells. IL-21 is a type I cytokine with effects on a number of cell types, including a non-redundant requirement in B cell activation and differentiation into plasma cells [3]. Levels of IL-21 are elevated in a number of animal models of lupus and also in human SLE. Blocking IL-21 is effective in animal models of lupus, whereas polymorphisms in both the IL-21 gene and in the IL-21 receptor gene are associated with human SLE. Trials of blocking IL-21 in human SLE have not yet begun. BAFF/BLyS(TNFSF13b) is a TNF family member that binds to 3 separate receptors and contributes to both naïve B cell and plasma cell survival [4]. Overexpression of BAFF/BLyS in mice leads to a lupus-like disorder, whereas blocking this cytokine ameliorates lupus in mouse models. Clinical trials in human SLE of a blocking monoclonal antibody, belimumab, have shown moderate clinical benefit associated with decreases in anti-DNA antibody titers and a decrease in circulating naïve B cells and plasma cells. These results led to the approval of this product for the treatment of SLE in the US. The approval of belimumab for treatment of SLE has confirmed that targeting B cells can be effective in treating this disease and has provided impetus for the development of additional B cell-directed therapies aimed at blocking cytokines involved in B cell survival and/or functional responsiveness.
